# Tobacco and hypertension: a descriptive study in a psychiatric short care unit

**DOI:** 10.1192/j.eurpsy.2023.2025

**Published:** 2023-07-19

**Authors:** I. Alonso Salas, A. Lopez Fariña, C. Gonzalez Navarro, A. Bilbao Idarraga, L. Morado San Segundo, U. Lopez Puentes, R. Lopez Brokate, T. Ruiz de Azua Aspizua, E. M. Garnica de Cos, U. Ortega Pozas, B. Samsó Martinez

**Affiliations:** Psychiatry, Red de Salud Mental de Vizcaya, Zamudio, Spain

## Abstract

**Introduction:**

Patients affected by mental disorders are known to have a decreased life expectancy.

One of the main reasons are cardiovascular diseases. It is known that tobacco and hypertension are risk factors to develop them. WHO estimates that hypertension is diagnosed and treated in less than half of adults with hypertension, and even less in patients with severe mental illness.

**Objectives:**

To describe the demographic characteristics of patients with tobacco comsumption and hypertension admitted to a short-term hospitalization unit.

**Methods:**

A three-month retrospective observational study. Data were collected by interviewing incoming patients and performing a blood pressure measurement, with no exclusion criteria.

**Results:**

Of 172 patients admitted, 100 were smokers of whom 49 were men and 51 were women. Among the smokers, a total of 18 patients were diagnosed with hypertension and 79 were not diagnosed. Within the group of patients not diagnosed with hypertension, elevated blood pressure was recorded in 5 of them. A total of 67 patients were non-smokers, 23 of whom were male and 44 female. Among the non-smokers, 19 were diagnosed with hypertension and 48 were not, despite which elevated blood pressure levels were recorded in 4 of them. No data were collected from 5 patients.

**Conclusions:**

The prevalence of smokers in our sample was 58%. The prevalence of patients diagnosed with hypertension was 21,51% which is coherent with the existent literature. We did not find a higher percentage of hypertensive patients among the smokers admitted. There were patients who suffered from hypertension and were not diagnosed or treated previously.

**Disclosure of Interest:**

None Declared

